# Health Literacy, Primary Care Health Care Providers, and Communication

**DOI:** 10.3928/24748307-20210529-01

**Published:** 2021-07

**Authors:** Shirly Mor-Anavy, Shahar Lev-Ari, Diane Levin-Zamir

## Abstract

**Background:**

Decision-makers and health professionals face challenges in providing quality medical services while optimizing diminishing resources. Health literacy is associated with health outcomes and health system costs and influences the way in which communication is managed in the health system.

**Objective:**

This study examined the association between the level of health literacy of service providers in the community, their awareness of health literacy, their attitudes toward health literacy promotion, and the way in which they communicate with patients with low health literacy.

**Methods:**

A cross-sectional analytic study was conducted among 50 physicians and 50 administrative staff members in community clinics of the Maccabi Health Maintenance Organization in Israel.

**Key Results:**

Significant positive associations were found (*p* < .05) between the level of health literacy, the attitudes toward health literacy promotion, and the degree to which special communication techniques were used when treating patients with low health literacy. Significant associations were found (*p* < .01) between the level of awareness, as well as the attitudes toward health literacy promotion and the degree to which communication techniques were applied. Higher health literacy is associated with more favorable attitudes toward health literacy promotion. Additionally, a significant positive association (*p* < .01) was found between the attitudes toward health literacy promotion and the use of communication techniques. No mediation was found among the research variables.

**Conclusions:**

To the best of our knowledge, this is the first study that examines health literacy among physicians. The results indicate gaps in the awareness of, and attitudes toward, health literacy among community health care providers, thus suggesting the need for developing and applying guidelines for improving efforts of health system providers regarding health literacy and for applying recommended tools for health communication. **[*HLRP: Health Literacy Research and Practice*. 2021;5(3):e194–e200.]**

**Plain Language Summary:**

This study examined the link between the health literacy of health care providers (e.g., physicians, service administrators), their awareness and attitudes toward health literacy promotion, and how they communicate with patients with low health literacy. The findings showed significant and positive relationships between these aspects of health literacy as well as gaps in the health care system that need to be addressed.

Finding the balance between maintaining quality medical services and the optimal use of diminishing resources constitutes present and future challenges for decision-makers and clinical health professionals. Cooperation between service providers and patients is necessary for the success of treatment interventions (Berkman et al., 2011; Castro et al., 2016). Interactive treatment protocols based on communication that encourage self-management and empowerment of patients have been proven efficient and economical (Castro et al., 2016). Health literacy is an essential component related to effective communication in the health system, with significant ramifications on people's health and health system costs (Franzen et al., 2014; Haun et al., 2015; Miller, 2016; Wu et al., 2016). As differentiated from health literacy needs in the hospital setting, health literacy in community settings requires communication that encourages and supports self-management of patients and their families in the long term (Nutbeam et al., 2018). Health literacy represents the cognitive and social skills that determine the motivation and ability of people to gain access to, understand, and use information in ways which promote and maintain good health (Nutbeam, 1998). To promote health literacy among patients and improve their health, it is necessary for service providers to acquire skills related to health literacy and to implement strategies including evaluation of health literacy and appropriate interventions. A systematic review by the Agency for Health Research and Quality found a consistent relationship between low health literacy and poor health outcomes (Berkman et al., 2004). Evidence shows that people who have low health literacy experience challenges in dealing with chronic health situations (Heijmans et al., 2015; Miller, 2016) and in navigating the health system (Bade et al., 2008). They have a higher risk for hospitalization (Cartwright, 2016) and increased risk of mortality (Baker et al., 2007) compared to those with higher health literacy. A systematic survey of 44 studies that examined the results of low health literacy found that people with a low level of health literacy had (1) less information regarding smoking, high blood pressure, diabetes, birth control, and limited understanding of instructions in a release letter from the emergency room; (2) worse morbidity measures including poor glycemic control among people with type 2 diabetes, fewer early detection examinations, and less preventive medicine; (3) excessive use of addictive substances; and (4) lack of skills for properly taking medication (DeWalt et al., 2004). In summary, low health literacy is associated with poor health and problematic use of health resources. People with low health literacy had a 1.5 to 3 times higher risk of suffering from a poor health condition compared to those with adequate health literacy (Parker, 2007; Schillinger et al., 2002).

Regarding the economic perspective, according to the Institute of Medicine (IOM), it was estimated that expenses for additional medical treatments attributed to low health literacy in the United States totaled $73 billion per year (Jukkala et al., 2009). Consequently, patients with low health literacy and their health care providers both pay higher medical costs due to their inefficient use of the health system (Eichler et al., 2009; Howard et al., 2005).

Although the U.S. health care system is different than the Israel health care system, IOM data provides an important perspective regarding the high cost of lower health literacy.

## Health Literacy Intervention in the Health System

Intervention related to increasing low health literacy has two parts. First, health care providers can be trained for raising awareness regarding health literacy, identifying needs, increasing individual knowledge, and improving beliefs while enhancing interpersonal and organizational communication. Second, patients can be empowered regarding self-awareness about health literacy skills and improving them (Castro et al., 2016; Parker & Ratzan, 2010). For health systems, interventions related to health literacy are often defined as patient-centered communication protocols and strategies. They include health literacy assessment during treatment or action taken to improve the level of health literacy, minimizing the negative repercussions of low health literacy. An online survey identifying health literacy interventions for improving provider communication to a patient with low health literacy during a clinical visit identified five different techniques: (1) group effort by medical staff beginning with the admission desk; (2) the use of standard health communication tools; (3) the use of a simple language during face-to-face interactions, supported by clear educational material; (4) cooperation between physician and patient for the purpose of defining treatment goals; and (5) organizational obligation for creating an environment that enables awareness and coping abilities with low health literacy (Barrett et al., 2008). These interventions are a promising approach to improving health-related outcomes; however, existing evidence indicates that many health literacy interventions are not routinely used by health care service providers. A study that examined the use of techniques for improving the level of health literacy through self-reporting among health care service providers (physicians, nurses, and pharmacists) found that the main techniques for dealing with patients with low health literacy were limited to the use of simple language, printed materials, and slow speech (Schwartzberg et al., 2007).

In the Israeli health care system, as in many others, the patient is required to integrate medical and administrative information to navigate health services. Service providers, whether clinical or administrative, may play a key role in health literacy promotion. To learn whether the service providers promote health literacy and/or communicate in accordance with the level of patients' health literacy, it is important to examine the awareness of service providers with regard to health literacy, the ramifications of low health literacy on patients' health, and the perceived financial expense incurred by health systems. Likewise, it is important to learn of their attitudes toward their responsibility for promoting health literacy and to discern the extent to which service providers promote health literacy and/or communicate in accordance with the level of health literacy among patients. Finally, the level of providers' health literacy may also provide a key to understanding their needs and how it may be associated with the action they take with their patients with low health literacy.

The objective of this study was to examine the association between the level of health literacy of health service providers, their awareness of health literacy, attitudes toward health literacy promotion, and the way in which they approach low health literacy among patients.

## Methods

### Study Design and Participants

A cross-sectional analytic study was conducted among a convenience sample of 50 physicians and 50 administrative staff members of the Maccabi Health Maintenance Organization in Israel between April and August 2017. The study focused on two main sectors responsible for providing patients with information on a daily basis in community clinics—physicians specialists and administrative service representatives who voluntarily agreed to participate in the study.

The study was approved by the Maccabi Health Service (using the Helsinki protocol) ethics committee (0146-16-asmc) as well as the Tel Aviv University ethics committee.

### The Study Measures

The level of health literacy was measured via a questionnaire, including 16 core questions from the European Health Literacy Survey measure validated in Israel (Levin-Zamir et al., 2016) using a 5-point scale. Five questions were added to adapt the tool to health care service providers in Israel to understand (1) the content of the medical documents that a patient signed prior to receiving medical care; (2) the brochure included in medicine packaging; (3) how to obtain information regarding rights as a patient; (4) how to obtain a screening examination appointment; and (5) nutritional food labeling. Participants were asked about their self-management within the health system, how difficult or easy it is for them to access/obtain health information, understand health information, process/appraise health information, and apply/use health information. Each question was equally weighted, thus the health literacy score ranged from 0 to 21. Reliability of the tool was tested using Cronbach's alpha (0.97). Providers' awareness regarding health and economic consequences of low health literacy described in the literature (Macabasco-O'Connell & Fry-Bowers, 2011) was measured via a 15-statement questionnaire, based on the U.S. National Institute of Medicine “Ten Attributes of a Health Literate Organization” model, (Brach et al., 2012), using a scale of 0 to 6. Attitudes toward health literacy promotion were based on the World Health Organization definition of health literacy (Nutbeam, 1998). Participants were asked which items related to their personal or their employer's role. Eight communication techniques were measured: (1) plain language, (2) speaking slowly, (3) with a clear voice, (4) use of written medical information with and (5) without any verbal explanation or demonstration, (6) asking questions to confirm patients understanding, (7) including repetition of treatment instructions, and (8) sequence of recommended actions. Use of communication techniques among patients with low health literacy was calculated based on the sum of self-reported responses on a scale of 0 to 6 (Schwartzberg et al., 2007). Initially, a pilot study was conducted (*N* = 30) among providers. Validity of the questionnaire was tested via Pearson's correlation coefficient examining linear relationships between consecutive variables. Reliability was tested via Cronbach's alpha measure. The score for health literacy awareness was 0.92, for attitudes was 0.84, and for communication technique use was 0.82. Secondly, a cross-sectional quantitative study was conducted based on the above-mentioned variables. Background variables included sociodemographic characteristics: gender (male/female); age (year of birth); religiosity (secular, traditional, religious, ultra-Orthodox), marital status, (single, married, separated, divorced, widowed), parental status (none, children age 0–18 years, children older than age 19 years); level of education (basic, small religious school [“yeshiva”], high school, academic); and occupation (physician, service administrator).

### Study Sample and Statistical Analysis

The sample size was calculated to measure associations between consecutive variables (*r* = 0.3) at a significance of 5% (*p* ≤ 0.05) and a power of 80%, which resulted in *N* = 86, with 43 participants in each test group. To test scale to the number of groups across variants, an additional 20% was added, for a sample size of approximately *N* = 100. Questionnaires were sent by email to 300 participants to reach the desired sample size. Consecutive data were described via averages and standard deviations, and categorical data were described via frequency and percentages. The distribution of the variables was examined via a Kolmogorov-Smirnov test. As the variables were not distributed normally, two non-parametric tests were used: Spearman correlation Mann-Whitney and Kruskal-Wallis. Chi-squared tests were applied for examining relationships between categorical variables. The health service providers' attitudes toward health literacy promotion as a mediating variable were analyzed via logistical hierarchical regression, controlling for age and gender.

## Results

### Sample Description

Among the physicians, 58% were women, the average age was 45 years (standard deviation [*SD*] = 7.36), 80% were secular, 60% were married, 100% were academics, and 46% had children between the ages of 0 and 18 years. Among the administrators, 100% were women, the average age was 41.5 years (*SD* = 10.58), 50% were secular, 52% were married, 60% had children between the ages of 0 and 18 years, and 44% had an academic degree. Thus, significant differences were found between the two groups regarding gender, religiosity, and education. The response rate was 50% among administrators and 25% among physicians.

### The Level of Health Literacy Among Service Providers

**Figure 1.**
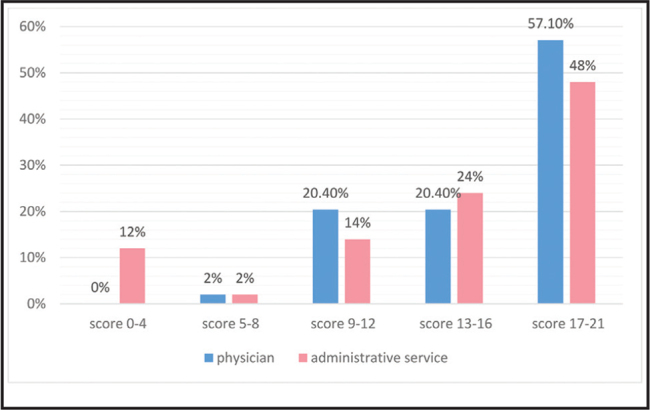
Distribution of health literacy.

Health literacy among providers was as follows: 52.5% high, 22.2% sufficient, 17.2% average, 2% problematic, and 6.1% inadequate. Additionally, 36% (22% administrators, 14% physicians) reported difficulty in understanding informed consent documents and 21% (13% administrators, 8% physicians) had difficulty in verifying where to receive medical treatment. The distribution of health literacy among the study participants is shown in **Figure 1**.

### Awareness and Attitudes Regarding Health Literacy Consequences

**Figure 2.**
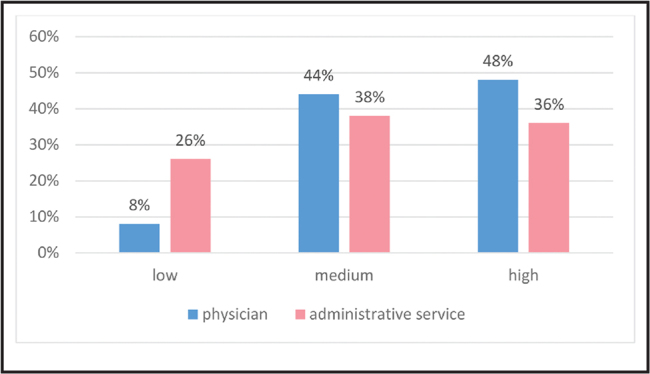
Level of awareness to the health and economic effects of low health literacy according to research groups.

The level of awareness among the providers regarding health and economic consequences of low health literacy was as follows: high among 42% of the service providers, medium among 41%, and low among 17% (**Figure 2**). One-third of the sample (22% of the administrators and 11% of physicians) were unaware that patients' low health literacy could lead to inappropriate use of the health care system. Similarly, 31% (18% administrators and 13% physicians) were unaware that low health literacy is related to higher health care costs. One-third of the providers, both administrators and physicians, were unaware that patients require skills in obtaining medical information and accessible communication channels in the health care system for making health decisions. Additionally, 22% of the providers (32% of the administrators, and 12% of the physicians) were unaware that patients with difficulties in understanding how to navigate the health care system are at risk for poorer health outcomes compared to patients skilled in navigating the system.

**Figure 3.**
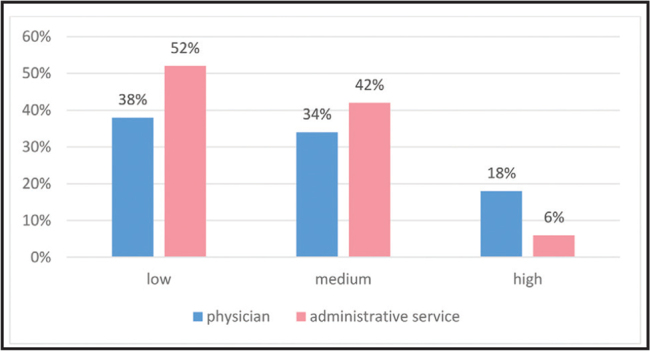
Service provider attitudes regarding their personal role in health literacy promotion according to research group.

Analysis showed that 67% of providers had never heard of health literacy, 26% had heard of the term but were not familiar with its meaning, and 7% had heard of the term health literacy and were well-versed with its meaning. The attitudes toward health literacy promotion among providers, including their role, was high only among 12%, medium among 38%, and low among 50% (**Figure 3**).

### The Use of Communication Techniques

**Figure 4.**
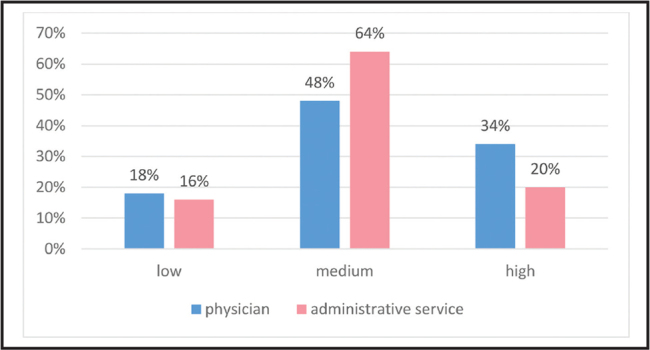
The use of communication techniques by service provider.

**Figure 5.**
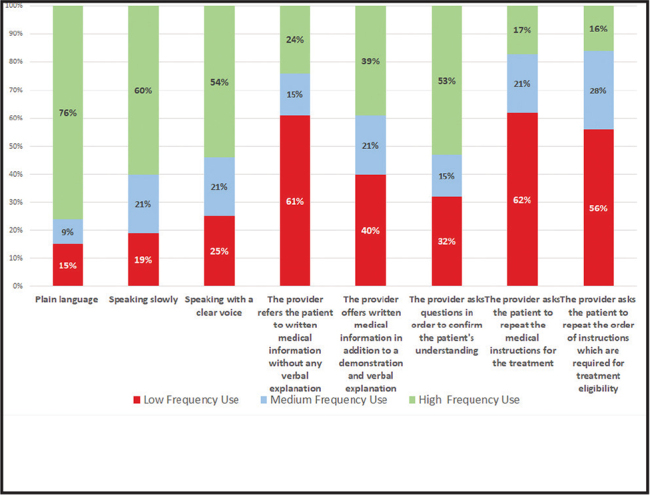
Distribution of reported use of specific communication techniques.

The reported use of tailored communication techniques with patients with low literacy was high among 27% of providers, medium among 56%, and low among 17% (**Figure 4**). The reported distribution of use of communication techniques by service providers to promote health literacy among patients is shown in **Figure 5**.

### Identifying Patients with Low Health Literacy

More than one-third (38%) of the sample reported having skills in identifying patients with low health literacy, 48% reported limited ability, and 14% reported the inability to identify low health literacy among patients; of these 14% of providers, 57% were physicians and 43% administrative staff.

### Associations Between the Research Variables

Significant positive associations were found (*p* < .05) between the level of health literacy, attitudes towards health literacy promotion, and reported use of communication techniques. The higher the level of health literacy, the more positive the attitudes toward health literacy promotion and the greater the use of appropriate communication techniques. Similarly, significant positive relationships were found (*p* < .01) between the level of awareness, attitudes towards health literacy promotion, and the degree of use of communication techniques. No mediating variables were found between the research variables.

## Discussion

This is the first study of its kind in Israel to explore health literacy among providers in the health care system. The population examined—clinical physicians who provide treatment information, and administrative staff who provide information on entitlement to services—are those from whom the patients are expected to receive information and instruction for promoting and maintaining health. Patients with low health literacy require support in acquiring and understanding information, partly from providers who themselves report limitations in accessing, understanding, and applying health information. Among the administrative staff, the prevalence of low health literacy is particularly significant (14%) when considering that it is expected of them to assist in integrating medical information with information on patients' entitlement to receive medical services. Additionally, although physicians' responses showed only 2% with low health literacy, in absolute numbers, a large number of physicians who provide community medical services have low health literacy. Low health literacy in this regard may reflect difficulty experienced in accessing and understanding relevant information on dynamic health topics such as navigation of the health care system, patients' rights, perceived reliable sources of health information, and more. Future studies may examine this issue more in-depth to understand the significance of low health literacy among professionals in the health care context. Regarding familiarity with the term health literacy, two-thirds of the participants reported never having heard of the term. This result is higher than the findings reported in the literature among nurses, where 80% reported familiarity with the term health literacy (Macabasco-O'Connell & Fry-Bowers, 2011). Additionally, our study revealed that only 42% of the providers showed a relatively high level of awareness of the effects of low health literacy. This may be compared to a survey conducted in the United States in which about 70% of respondents were aware of the effects of low literacy levels (Brown et al., 2004). The noted difference may be due in part to the difference in methods of measurement. As other researchers suggest, a lack of estimation of a patient's level of health literacy may be an important source of health disparities (Kelly & Haidet, 2007). In our study, the low level (38%) of reported skills demonstrates the need for empowering providers with skills in understanding their patients' level of health literacy.

We found that 50% of providers perceived having a high to medium level of personal responsibility regarding their role in health literacy. We learned that understanding the importance of recognizing low health literacy among patients was associated with awareness of its effects, as well as with the providers' positive attitude toward their personal role in promoting health literacy. We also found that only 16% of study participants reported using communication techniques on a daily basis, which is similar to the numbers mentioned in the literature (Barrett et al., 2008). This finding indicates another gap in the health care system. Accordingly, the results indicate the need for further research to explore how improving health literacy among providers could ultimately lead to empowerment and improvement of patients' health literacy.

## Study Limitations

Although the study contributes to the existing body of knowledge, it was tested only among physicians and administrators in one region and organization in Israel; therefore, the results cannot be generalized to providers in the entire health care system. Additionally, the data collected were based on self-reporting that may be affected by recall bias, which we tried to reduce by asking participants to report on the most recent period. Also, with regard to the subjective nature of the health literacy instrument, there may be concerns regarding predictive validity. We did not test the participants for validating the difficulty or ease with which they estimated that they are capable of performing the relevant tasks included in the research tool. To note, a significant correlation was found between the level of health literacy and reported communication techniques among service providers. Future studies may focus more specifically on the issue of predictive validity. Finally, as a cross-sectional study, causality cannot be determined, so future prospective studies are warranted to assess causality between variables.

## Practice Implications

In the Israeli health care system, each individual person is required to integrate medical and administrative information, for navigation and receiving medical and administrative services. The findings of the study show that a considerable rate of providers reported insufficient health literacy, which could affect their ability to support their patients with low health literacy, including the use of effective communication techniques. Moreover, the results of this study may provide a basis for improving providers' attitudes toward health literacy, expanding their role in improving health literacy, and, in turn, promoting community health.

## Conclusions

To the best of our knowledge, this is the first study to examine health literacy among physicians. The results indicate gaps in the field of health literacy regarding the level of health literacy among providers and their awareness and coping capabilities with low health literacy among patients. The results show the need for developing interventions to improve the collective effort of providers in the health care system and for promoting health literacy, including recommended tools and techniques for effective health communication.
